# Impacts of Climate Change on the Biogeography and Ecological Structure of *Zelkova schneideriana* Hand.-Mazz. in China

**DOI:** 10.3390/plants13060798

**Published:** 2024-03-11

**Authors:** Chen Wang, Yuanlan Zhang, Qianqian Sheng, Zunling Zhu

**Affiliations:** 1 College of Landscape Architecture, Nanjing Forestry University, Nanjing 210037, China; wangchen1999@njfu.edu.cn; 2 Co-Innovation Center for Sustainable Forestry in Southern China, Nanjing Forestry University, Nanjing 210037, China; 3 Research Center for Digital Innovation Design, Nanjing Forestry University, Nanjing 210037, China; 4 Jin Pu Research Institute, Nanjing Forestry University, Nanjing 210037, China; 5 College of Life Sciences, Nanjing Forestry University, Nanjing 210037, China; 6 College of Art and Design, Nanjing Forestry University, Nanjing 210037, China

**Keywords:** *Zelkova schneideriana*, climate change, land-use change, species distribution model, landscape fragmentation

## Abstract

This study utilized the platform for ensemble forecasting of species distributions, biomod2, to predict and quantitatively analyze the distribution changes of *Zelkova schneideriana* Hand.-Mazz. under different climate scenarios (SSP1-2.6 and SSP5-8.5) based on climate and land-use data. This study evaluated the geographic range changes in future distribution areas and the results indicated that, under both SSP1-2.6 and SSP5-8.5 scenarios, the distribution area of *Zelkova schneideriana* would be reduced, showing a trend towards migration to higher latitudes and elevations. Particularly, in the more extreme SSP5-8.5 scenario, the contraction of the distribution area was more pronounced, accompanied by more significant migration characteristics. Furthermore, the ecological structure within the distribution area of *Zelkova schneideriana* also experienced significant changes, with an increasing degree of fragmentation. The variables of Bio6 (minimum temperature of the coldest month), Bio2 (mean diurnal temperature range), Bio15 (precipitation seasonality), and elevation exhibited important influences on the distribution of *Zelkova schneideriana*, with temperature being particularly significant. Changes in land use, especially the conversion of cropland, had a significant impact on the species’ habitat. These research findings highlight the distributional pressures faced by *Zelkova schneideriana* in the future, emphasizing the crucial need for targeted conservation measures to protect this species and similar organisms.

## 1. Introduction

Understanding the dynamic changes in species distribution is crucial for biodiversity conservation and ecosystem management in the context of global climate change [1]. Climate change is one of the main driving forces behind species distribution and biodiversity change [2,3,4], and it has profound impacts on the structure, function, and ecological traits of species [5,6,7]. Climate change will alter the suitable habitats for plants, with some plants showing spatial distribution patterns that involve migrating towards higher latitudes and elevations to track their ecological niches [8,9,10]. However, some studies have also found that certain plants migrate towards lower elevations and latitudes to adapt to changing conditions [11]. The IPBES (Intergovernmental Science-Policy Platform for Biodiversity and Ecosystem Services) global assessment report indicates that land-use change is a significant factor threatening species survival, as it causes landscape changes and fragmentation of distribution areas, leading to habitat degradation, habitat isolation, and disruption of species interactions [12,13]. These changes ultimately determine the abundance, occurrence, and connectivity of biological populations [14]. Research has found that climate change and land-use change will collectively impact biodiversity and geographical distribution patterns [15,16,17].

*Zelkova schneideriana* Hand.-Mazz., a woody plant of the Ulmaceae family and the *Zelkova* genus, is characterized by its dense, durable qualities and aesthetically pleasing grain patterns, rendering it suitable for premium hardwood applications. Furthermore, its broad canopy, graceful silhouette, and rich seasonal foliage variations make it a preferred choice for ornamental landscaping purposes [18,19,20]. *Zelkova schneideriana* is primarily distributed in the subtropical regions of China [21]. Research conducted by Shao and Zhang on the *Zelkova schneideriana* communities in Jiangsu, China, demonstrated that *Zelkova schneideriana* is the most important dominant species, exhibiting limited competitive interactions with other tree species [22]. Building upon this, research conducted by Zhou et al. revealed that the *Zelkova schneideriana* predominantly occurs in two forest ecosystems within China’s subtropical regions: deciduous broad-leaved forests and mixed bamboo and broad-leaved forests, covering 19 provinces nationwide [23]. However, excessive human logging and weak natural regeneration capacity have resulted in a continuous decrease in its distribution [24,25]. Currently, it is classified as a second-level protected wild plant in China and considered a vulnerable species in the IUCN Red List for China (https://www.iucnredlist.org/species/131155456/131155458 (accessed on 11 December 2023)). In recent years, the changes in the distribution range of *Zelkova schneideriana* have received focused attention due to its value and rarity. Research conducted by Sun et al. suggested that human activity intensity and future climate change will cause a northward shift in the distribution of *Zelkova schneideriana*, leading to a reduction in the distribution area. Precipitation was identified as the main factor influencing its distribution [26]. Additionally, Zhou et al. conducted a further study on the changes in the distribution range of *Zelkova schneideriana* based on more accurate records of its natural distribution. The results similarly indicated a northward shift in the distribution range of *Zelkova schneideriana* in the future, with temperature identified as the dominant factor affecting the distribution change [23]. However, these studies did not consider the impact of land utilization changes on the distribution of *Zelkova schneideriana*, nor did they explore the changes in the internal structure of the distribution area.

To comprehensively assess the potential risks of future distribution changes in *Zelkova schneideriana*, it is necessary to not only study the integrated impacts of climate and land-use changes [27] but also consider the spatial characteristics of the distribution area’s internal structure. The use of large-scale indicators that characterize geographical distribution changes cannot accurately evaluate their responses to climate and land-use changes [28]. Currently, landscape indicators have been developed and applied to comprehensively capture the structural changes of species within their distribution areas, thereby aiding in the elucidation of species’ ecological responses within complex landscapes [17,29].

Therefore, this study aims to (1) predict the distribution range of *Zelkova schneideriana* under future climate scenarios based on climate change and land-use data. (2) Quantify the internal structural changes within the distribution area of *Zelkova schneideriana*. (3) Evaluate the differences in distribution area changes of *Zelkova schneideriana* under different climate scenarios. (4) Analyze the factors influencing the changes in the distribution area of *Zelkova schneideriana.*

## 2. Results

### 2.1. Accuracy of the Model’s Predicted Results

The research results indicate that the optimal TSS (True Skill Statistic) value for the TSS threshold model is 0.805, and the AUC (Area Under Curve) value is 0.956 (Table 1). These values, exceeding 0.8, demonstrate the high predictive accuracy of the ensemble model for the distribution range of *Zelkova schneideriana*. Furthermore, the model effectively simulates the distribution of *Zelkova schneideriana* under climate change conditions.

### 2.2. Changes in the Distribution Area of Zelkova schneideriana

**Figure 1:**
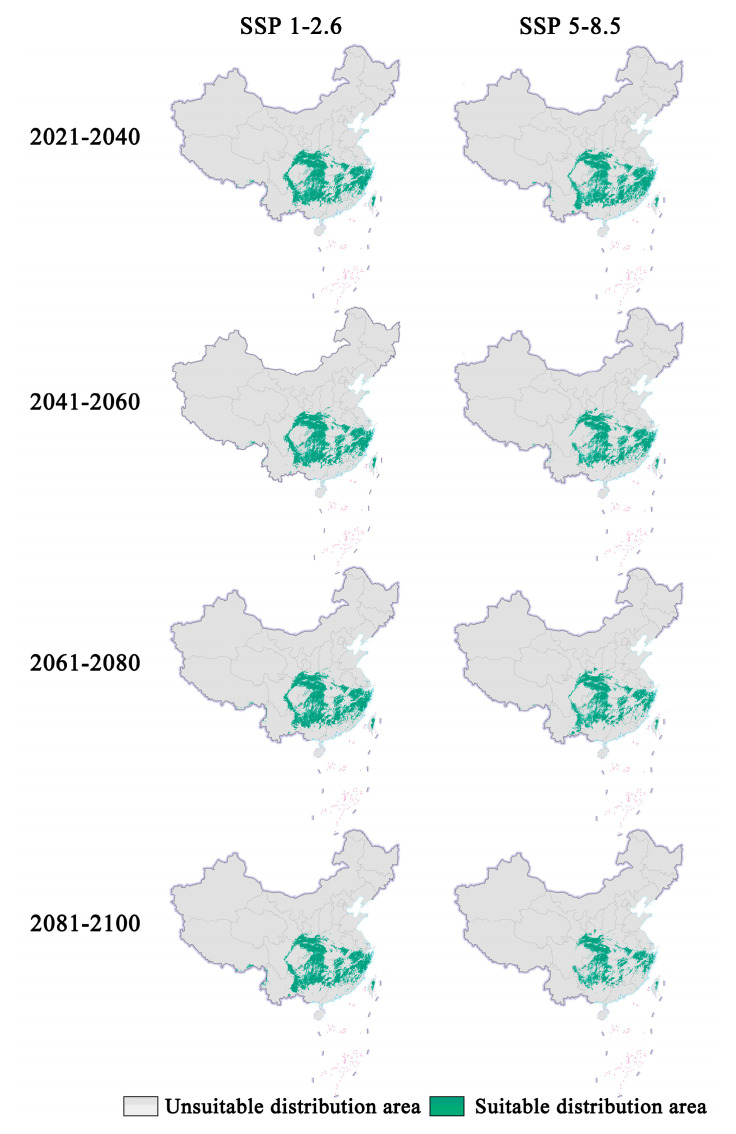
Prediction of future distribution areas of *Zelkova schneideriana* under different climate change scenarios in China. (Based on the standard map production of the Ministry of Natural Resources of the People’s Republic of China (bzdt.ch.mnr.gov.cn (accessed on 1 March 2024)), GS (2023) 2763; the base map boundaries remain unaltered. The same applies below.).

**Figure 2:**
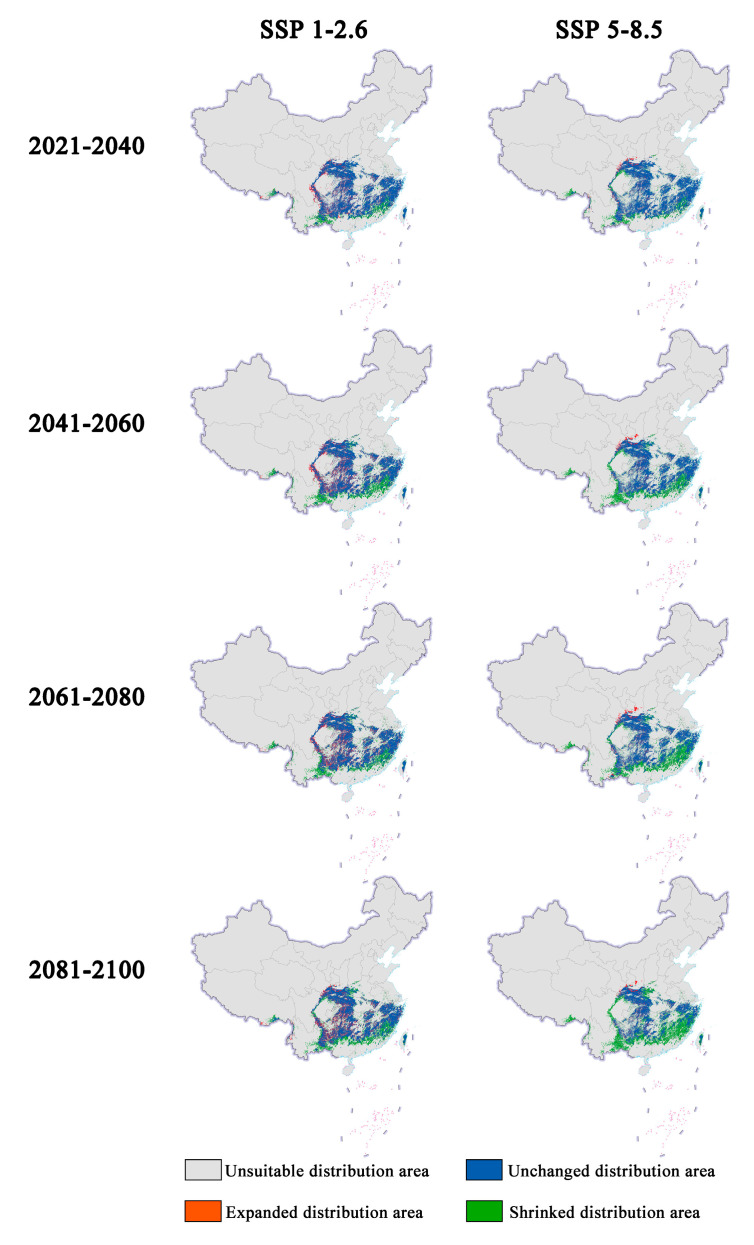
Changes in future distribution areas of *Zelkova schneideriana* under different climate change scenarios in China.

**Figure 3:**
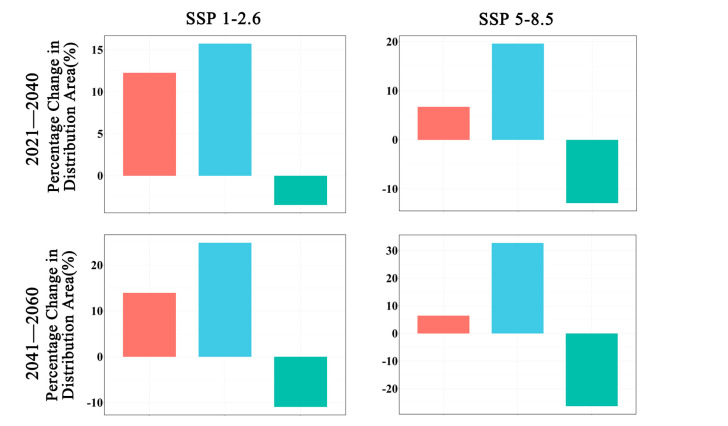

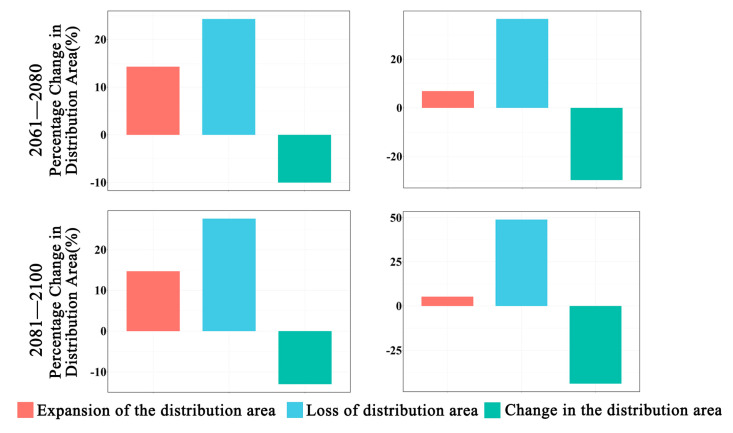
Changes in the distribution area of *Zelkova schneideriana*.

In future climate scenarios, the distribution area of *Zelkova schneideriana* is predicted to contract (Figure 1, Figure 2 and Figure 3). Under the SSP1-2.6 scenario, the contraction trend is relatively moderate, with a noticeable expansion (>10%) and contraction (>15%) of the distribution range. However, under the SSP5 8.5 scenario, the changes in the distribution area are more severe. Compared to the SSP1-2.6 scenario, the expansion trend is slowed (<10%), while the contraction trend intensifies. By 2081, the contraction is projected to reach close to 50%, posing a severe issue for the distribution of *Zelkova schneideriana*.

### 2.3. Changes in the Latitude and Elevation of the Distribution Area of Zelkova schneideriana

**Figure 4:**
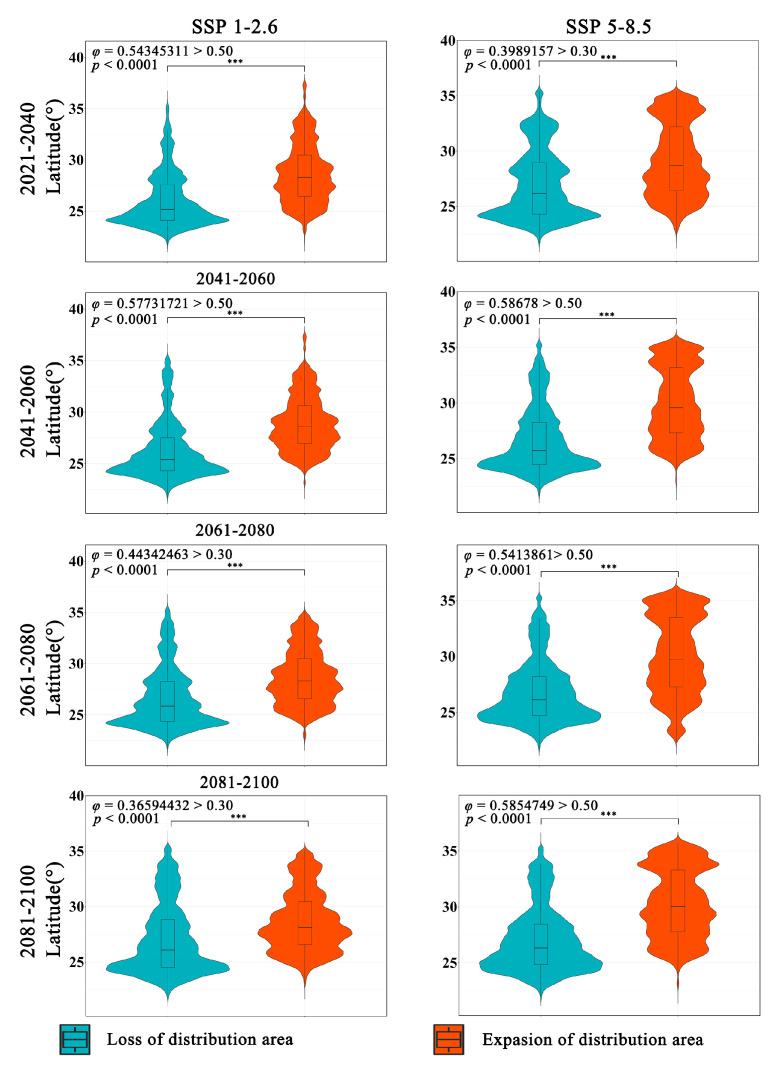
Differences in latitude variations in the distribution area of *Zelkova schneideriana*. (The *p* value significance codes: “***”: *p* < 0.001. The same applies below.)

The latitudinal range of the expansion and contraction of the distribution of *Zelkova schneideriana* varies significantly regardless of climate scenarios (Figure 4). The contraction of *Zelkova schneideriana* distribution is concentrated in lower latitudes, with an increasing density of distribution at lower latitudes as time progresses. Conversely, the expansion area shows the opposite trend, with a higher density of distribution concentrated at higher latitudes and an increasing density of distribution at higher latitudes as time progresses. The differences between the SSP5-8.5 climate scenario and the SSP1-2.6 climate scenario are more pronounced in terms of distribution. Also, the latitudinal distribution density is not uniform. Both the statistically significant test (*p* < 0.0001) and the effect size measurement (*φ* > 0.3) indicate that this variation is not only statistically significant but also ecologically important.

**Figure 5:**
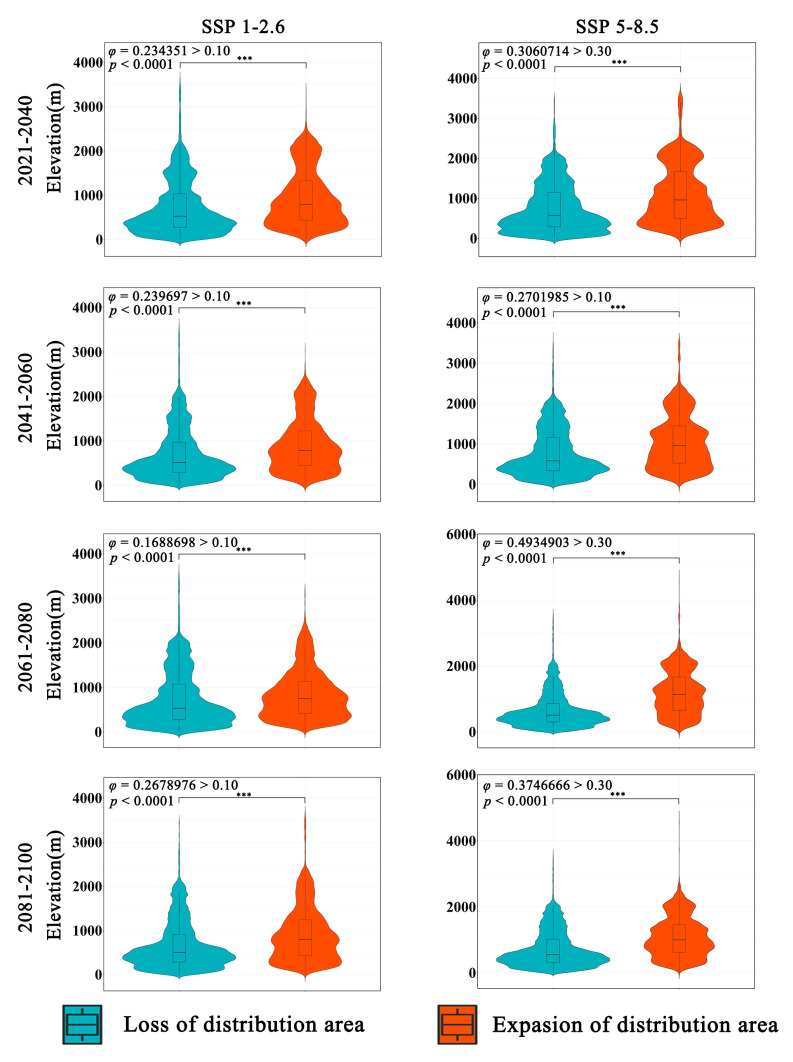
Differences in elevation variations in the distribution area of *Zelkova schneideriana*. “***”: *p* < 0.001.

The overall trend of *Zelkova schneideriana*’s elevational changes in two climate scenarios is consistent (Figure 5). The expansion area has a higher distribution density at higher elevations compared to the contraction area. A statistically significant test (*p* < 0.0001) indicates that this change is statistically significant. However, the effect size of the SSP1-2.6 climate scenario is low (*φ* > 0.1), suggesting a mild distribution change. On the other hand, the SSP5-8.5 climate scenario has a higher effect size compared to SSP1-2.6, indicating a more pronounced tendency for *Zelkova schneideriana* to shift to higher elevations. The violin plots of the expansion and contraction areas in both scenarios exhibit clear elongated tails at higher elevations, indicating greater variability in the distribution at higher elevations.

### 2.4. Internal Structural Changes in the Distribution Area of Zelkova schneideriana

**Figure 6:**
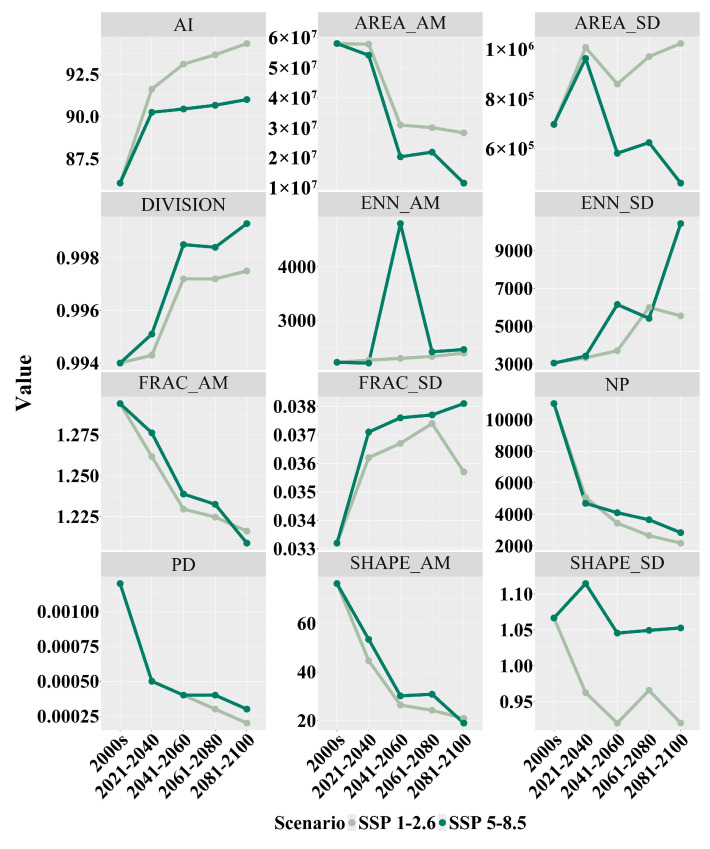
Landscape index changes in the distribution area of *Zelkova schneideriana*.

After studying the landscape index changes in the distribution area of *Zelkova schneideriana* in two scenarios, we found a series of structural changes in the distribution area of *Zelkova schneideriana* (Figure 6). An increase in the aggregation index (AI) was observed in both SSP1-2.6 and SSP5-8.5 scenarios, indicating a tendency for overall patch aggregation. SSP1-2.6 showed a higher aggregation index (AI) compared to the SSP5-8.5 climate scenario. While there was some improvement in local aggregation, the increase in landscape division index (DIVISION) revealed an overall increase in isolation between patches, suggesting the potential existence of barriers to movement between patch populations. The SSP5-8.5 climate scenario exhibited greater landscape fragmentation. Additionally, the significant peak observed in the area-weighted mean euclidean nearest-neighbor distance (ENN_AM) in the SSP5-8.5 scenario, as well as the stable increase in the SSP1-2.6 scenario, indicated increased patch separation. The upward trend in the standard deviation of the euclidean nearest-neighbor (ENN_SD) distance further highlighted the inconsistency in patch distances, suggesting that some patches in the habitat are more isolated compared to others. The continuous decline in patch number (NP) and the decrease in patch density (PD) in both scenarios further confirmed the increasing habitat fragmentation. The scenarios also showed a decreasing trend in the area-weighted mean shape index (SHAPE_AM) and the standard deviation of the shape index (SHAPE_SD), indicating a gradual simplification and regularity in the shape of the habitat patches and an increase in their consistency. The area-weighted mean fractal dimension index (FRAC_AM) displayed a decreasing trend in both scenarios, reflecting a tendency towards simplification and regularity in the shape of the patches, while the fluctuations in the standard deviation of the fractal dimension index (FRAC_SD) demonstrated an increasing inconsistency in patch shape and edge complexity between patches. The decrease in the area-weighted mean area (AREA_AM) indicated a reduction in the average size of the habitat patches, while the increase in the standard deviation of the area (AREA_SD) indicated an increasing inconsistency in the sizes of the habitat patches.

### 2.5. Factors Influencing Changes in the Distribution Area of Zelkova schneideriana

**Figure 7:**
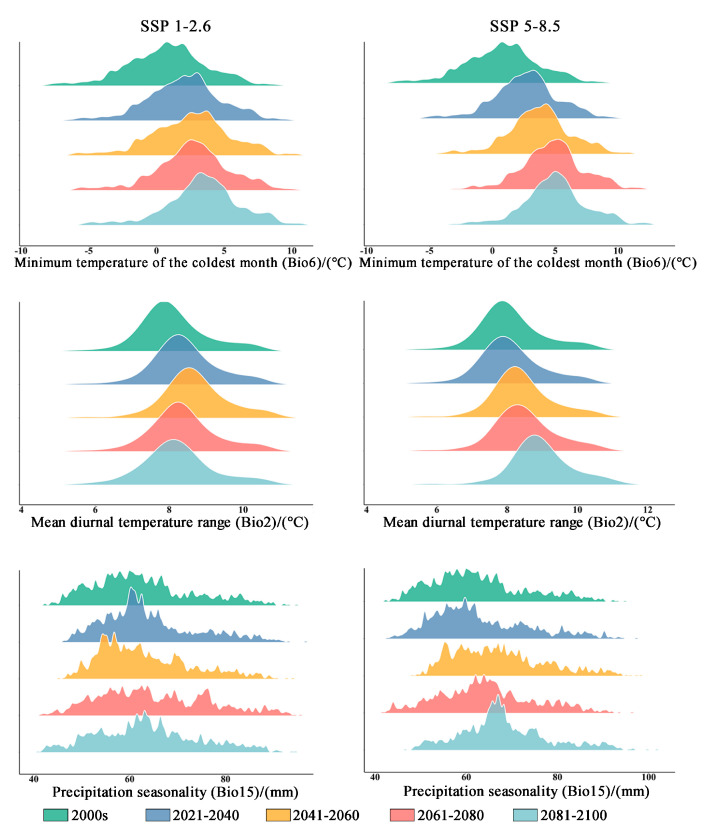
The bioclimatic conditions of the current distribution area of *Zelkova schneideriana*.

The results presented in Table 2 demonstrate that the contribution rate of Bio6 (minimum temperature of the coldest month) is the highest. Following this, Bio2 (mean diurnal temperature range) contributes the second-highest percentage. In comparison to elevation and precipitation, temperature factors have a higher contribution rate (79%). Under both climate scenarios, it is expected that Bio6 will experience a significant increase. The mean minimum temperature of the coldest month in the original distribution area is projected to rise from 0.769 °C to 3.449 °C (in the SSP1-2.6 climate scenario) and to 4.985 °C (in the SSP5-8.5 climate scenario) during the period from 2081 to 2100 (Table 3 and Figure 7). Additionally, the average Bio6 (mean diurnal temperature range) exhibits an increasing trend within the original distribution area, while the Bio15 (precipitation seasonality) shows an upward trend accompanied by greater variability (Table 3 and Figure 5).

**Figure 8:**
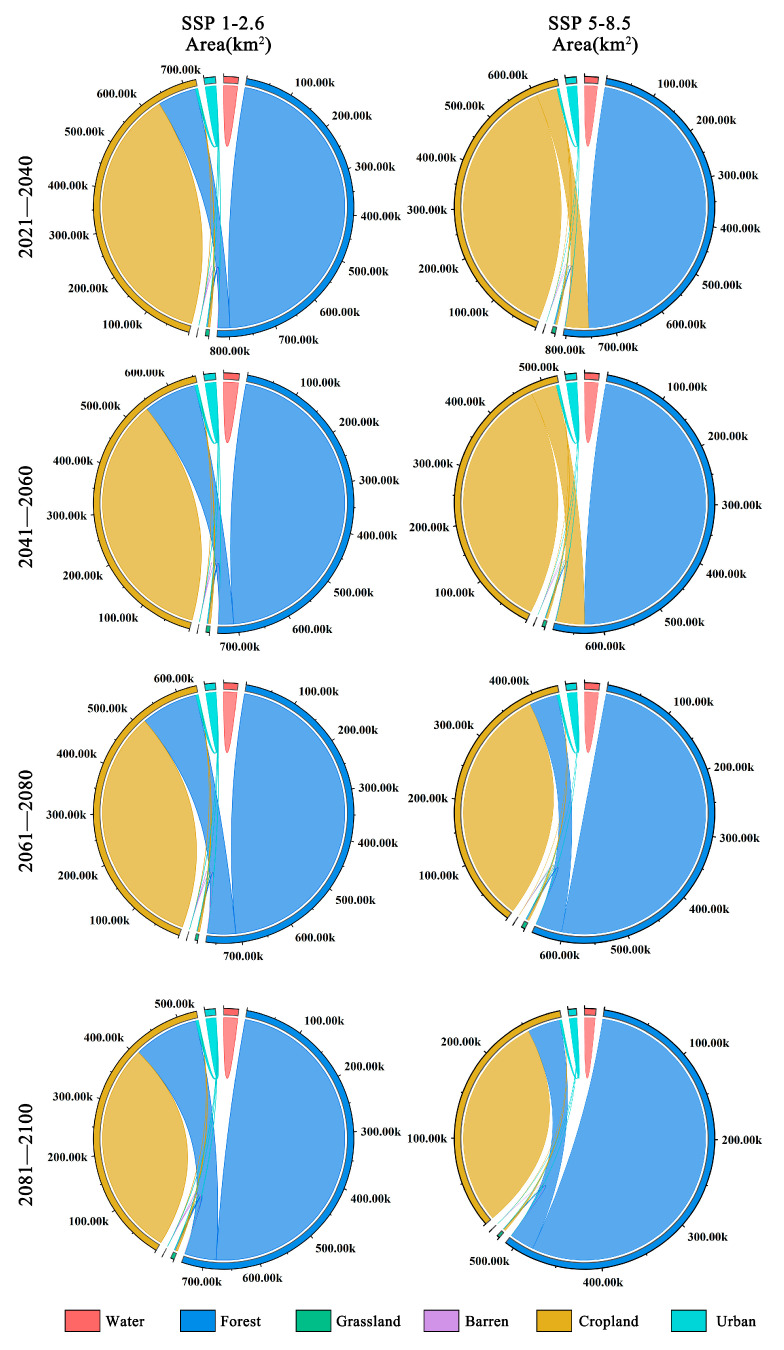
Future land-use changes in the distribution area of *Zelkova schneideriana*.

There are significant differences between the two scenarios of land-use change (Figure 8). Under the SSP1-2.6 climate scenario, land-use change is minimal due to human efforts in environmental conservation, leading to a gradual increase in forest area within the distribution area of *Zelkova schneideriana*. However, under the SSP5-8.5 climate scenario, from 2021 to 2060, we observed a substantial conversion of forest land into cropland within the distribution area of *Zelkova schneideriana*, accounting for over 50% of the total area. However, this trend reverses from 2061 to 2100, as cropland reverts back to forest land, resulting in the recovery of forest land, once again surpassing 50%.

## 3. Discussion

### 3.1. Factors Influencing the Distribution Area of Zelkova schneideriana

The geographic distribution of plant species is an important spatial characteristic that can be influenced by climate change, human activities, and other factors. Climate and land use are crucial factors affecting species distribution, and their levels of change are directly related to the patterns of species distribution and biodiversity. Our research findings indicate that temperature is the most influential factor affecting the distribution of *Zelkova schneideriana*. Among all climate variables, Bio6 (minimum temperature of the coldest month) and Bio2 (mean diurnal temperature range) have the greatest impact on the distribution of *Zelkova schneideriana*. This finding aligns with Zhou et al.’s previous study [23]. Zhou et al. suggested that the ample precipitation in subtropical regions is unlikely to be a limiting factor for the distribution of *Zelkova schneideriana*. Our research results confirm this assertion, as the Bio15 (precipitation seasonality) is not pronounced in the original distribution area. Furthermore, many studies on the distribution of subtropical plants in China have indicated that temperature may be the primary factor driving the distribution of plants in this region. Our previous research has shown that temperature dominates the distribution of landscape plants in both northern and southern regions [30]. However, this is not absolute. Research on *Pinus massoniana* Lamb. suggested that precipitation is the dominant factor, and Sun et al.’s research on the richness distribution pattern of oaks (*Quercus* L.) showed that annual precipitation (Bio12) exhibited the highest quantile compared with the other variables [31,32]. Therefore, the impact of climatic factors on the distribution of subtropical plants in China is heterogeneous, and further research is needed to understand the specific patterns.

Furthermore, our research proves the serious impact of land-use changes on the species distribution range. The high proportion of non-forested areas in the distribution area has resulted in the loss of suitable habitats for *Zelkova schneideriana,* and the most significant impact comes from cropland. Related studies have also shown that the impact of land-use changes on plant distribution cannot be ignored. For example, research by Song et al. on the extinction risk of eastern Asian plants indicated that the expansion of cropland and non-forest land will pose significant threats to plant distribution in the region, with cropland playing a prominent role [16]. Our research findings indicated that different development scenarios will have a significant impact on the survival status of *Zelkova schneideriana*. Under the SSP1-2.6 scenario, the conversion of cropland to forest mitigates the rate of disappearance of *Zelkova schneideriana* distribution areas, while under the SSP5-8.5 scenario, facing greater pressure from climate and land-use changes, the trend of *Zelkova schneideriana* distribution area loss further intensifies. Wang et al.’s study on coastal mangrove habitat yielded similar conclusions, suggesting that land-use policies aimed at mangrove conservation will effectively slow down the trend of habitat degradation in mangrove distribution areas [33].

### 3.2. Range Changes of the Distribution Area of Zelkova schneideriana

Over time, the distribution range of *Zelkova schneideriana* in both climate scenarios is expected to face contraction, consistent with previous findings [23,26]. In subtropical regions of China, variations exist in the trends of changes observed in plant distribution areas. Research findings regarding species such as *Litsea cubeba* (Lour.) Pers. and *Toona ciliata* Roem. suggested an expansion of their distribution ranges [34,35]. Conversely, studies on plants like some species of oaks (*Quercus* L.) were consistent with our own research [32], indicating a reduction in their future distribution areas. Additionally, our study reveals a potential expansion of *Zelkova schneideriana*’s distribution range towards higher latitudes and elevations, accompanied by a reduction in lower latitudes and elevations areas. This trend is expected to become more significant with intensifying climate warming, aligning with previous research [23,26]. The observed migration pattern may be related to changes in the species’ climatic niche. Over time, Bio6 (minimum temperature of the coldest month) and Bio2 (mean diurnal temperature range) in the original distribution areas are projected to increase. Consequently, *Zelkova schneideriana* tracks cooler climates by advancing towards the northern edge and contracting in the south. Similar conclusions have been drawn in related studies [8,36,37]. It is important to note that species distribution models assume unobstructed dispersal pathways, which may not be the case due to landscape barriers and *Zelkova schneideriana*’s limited dispersal capacity [8].

Furthermore, we observe that there is a higher effect value for the migration trend of the *Zelkova schneideriana* distribution range towards higher latitudes compared to elevation. This suggests that the latitude variation in the distribution range of *Zelkova schneideriana* is more pronounced. We hypothesize that the reason for this trend disparity is due to varying regional rates of climate change. Previous research has indicated that the rate of global warming is faster in flat areas at higher latitudes [38]. Additionally, since temperature is the dominant factor influencing the distribution of *Zelkova schneideriana*, it is expected that the distribution range will primarily shift towards higher latitudes.

In light of the shifting distribution patterns of *Zelkova schneideriana*, our foremost priority is to safeguard their stable natural habitats effectively, thereby preventing illicit logging and ecological degradation. Moreover, we advocate for heightened monitoring and investigative measures in regions vulnerable to habitat loss and expansion. Consistent monitoring and evaluation of population size, distribution range, growth status, and potential threats to *Zelkova schneideriana* are imperative for prompt issue detection and the implementation of suitable conservation strategies.

### 3.3. Fragmentation of Distribution Range Landscape

The results of the landscape index reveal a concerning trend of landscape fragmentation in the distribution areas of *Zelkova schneideriana* under two climate scenarios. In the SSP5-8.5 scenario, habitat fragmentation becomes more pronounced. The synergy between landscape fragmentation and climate change will decrease the species’ ability to adapt to climate change. Firstly, *Zelkova schneideriana* produces small fruit that relies primarily on gravity and wind for dispersal [39]. Consequently, the limited dispersal distance in fragmented landscapes hampers the species’ migration to habitats with the most suitable climatic conditions, resulting in restricted natural regeneration and impacting reproductive, dispersal, and persistence mechanisms [40,41]. Additionally, research has shown that *Zelkova schneideriana* exhibits a high chance of inbreeding due to its naturally small population size [39]. In turn, landscape fragmentation increases the likelihood of random genetic drift and inbreeding, leading to a reduced level of gene flow between populations. Self-pollinating species often exhibit lower levels of within-species genetic variation, which affects gene flow between individuals and populations and undermines their ability to adapt to changing climates [42,43,44]. Related studies have also reached similar conclusions. Investigations into the distribution patterns of species like *Tilia amurensis* Rupr. and *Phellodendron amurense* Rupr. in northeastern China have revealed instances of habitat fragmentation within regional plant habitats. As landscapes progressively transition towards human-dominated land use, the spatial cohesion of plant habitats may continue to diminish [17,45].

To safeguard and capitalize on the genetic diversity within *Zelkova schneideriana* populations while mitigating the effects of landscape fragmentation, we advocate for the prompt collection of wild germplasm resources from high-quality wild *Zelkova schneideriana* tree communities. Assessing their diversity will lay the groundwork for future cultivation and conservation endeavors.

## 4. Materials and Methods

### 4.1. Data Collection

The distribution data of *Zelkova schneideriana* were obtained from the dataset provided by Zhou et al. which offers detailed records of *Zelkova schneideriana* distribution [23]. A total of 312 specimen points of *Zelkova schneideriana* were used for modeling purposes. For more detailed information on the methods of data collection and processing, please refer to Zhou et al. [23].

The climatic and elevation data used for modeling in this study were obtained from the WorldClim database (https://worldclim.org/). Climate data from the SSP1-2.6 and SSP5-8.5 scenarios under the BCC-CSM2-MR model were selected. The resolution for all data was 2.5 arc-minute. The BCC-CSM2-MR model is a medium-resolution climate system model developed by the National Climate Center. It exhibits improved performance in simulating future climates for China compared to its earlier version, BCC-CSM-1.1m [46,47]. Furthermore, compared to CMIP5, SSPs provide a better reflection of the relationship between socioeconomic development and climate scenarios [48].

In the context of the SSP-RCP scenarios, we utilized a high-resolution (1 km) land-use dataset developed by Chen et al. [49]. This dataset encompasses seven key land types, namely, water, forest, grassland, barren, cropland, urban, and permanent snow and ice. The inclusion of these data augments our understanding of the impact of anthropogenic changes on the Earth’s surface over time.

**Figure 9:**
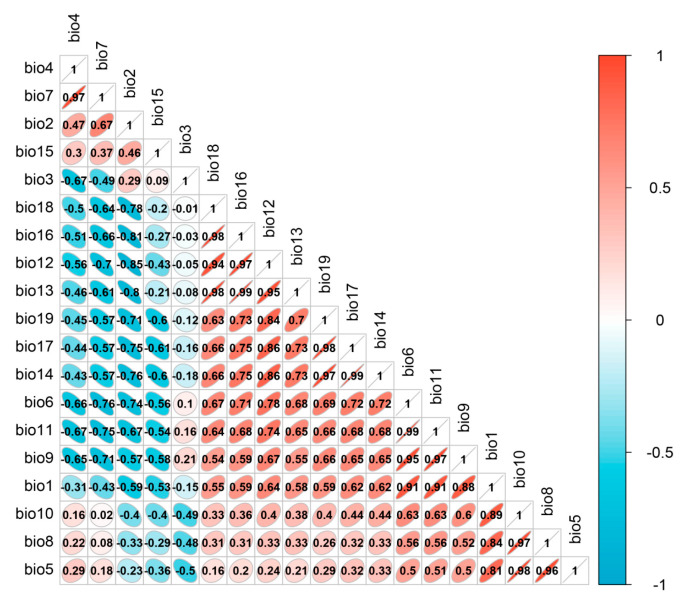
Results of the correlation analysis of bioclimatic variables.

Due to the correlation among the 19 bioclimatic variables (Figure 9), using all of them in the model predictions may lead to overfitting. Consequently, this study screened the data of the 19 bioclimatic variables. Firstly, a correlation analysis was performed on the 19 climate variables. Then, an ensemble model was employed to model the 19 climate variables, yielding the important results for each variable. Variables with a correlation coefficient greater than 0.75 were considered highly correlated, and only those with high importance and interpretability were retained for species distribution modeling. The method of evaluating and selecting variables based on their importance using ensemble models allows for a more comprehensive consideration of each variable’s contribution to model predictions compared to other approaches for addressing multicollinearity [50,51]. Consequently, this method facilitates the selection of important variables, thereby enhancing the interpretability of the model. The climate variable data, digital elevation data, and land-use data used for modeling were standardized to a spatial resolution of 1 km using the resampling tool in ArcGIS 10.8.1.

### 4.2. Species Distribution Model

This study employed an ensemble modeling approach known as presence–absence modeling, which consisted of four modeling methods: Generalized Linear Models (GLMs) [52], Generalized Boosted Models (GBMs) [53], Maximum Entropy Models (MaxEnt) [54], and Random Forest (RF) [55]. Default parameters were used for all models. The use of multiple modeling techniques can reduce the prediction bias caused by individual models, and ensemble modeling has been widely used in species distribution prediction [16,51]. The data were divided into training data (75%) and testing data (25%) [56]. We generated pseudo-absences in the same quantity as the *Zelkova schneideriana* specimen points to reduce uncertainty introduced by random sampling. Three sets of pseudo-absences were generated to balance the omission and commission errors in model predictions, and each algorithm was repeated 10 times [57]. All models and ensemble predictions were performed using the R 4.2.3 package “biomod2” [58].

We employed True Skill Statistics (TSS) [59] and the area under the Receiver Operating Characteristic curve (AUC) for model evaluation. AUC is not always sufficient to consider model fitness or to penalize excessive predictions [60]. Additionally, the median, being robust to outliers, is insensitive to abnormal values. Hence, in this study, we utilized a median ensemble based on TSS.

### 4.3. Analysis of Factors Influencing Changes in Distribution Range

To investigate the climate factors influencing the distribution of *Zelkova schneideriana*, our study randomly selected 1000 pixels within the current species’ distribution area and computed the density (frequency), mean, and 95% quantile of the four variables [61]. Additionally, to characterize the impact of land-use change on the distribution of *Zelkova schneideriana*, our study calculated the changes in land use compared to the current scenario within the study area using the future distribution areas.

### 4.4. Analysis of Changes in Distribution Range

To quantify the changes in the distribution range of *Zelkova schneideriana*, we first generated a binary map based on habitat suitability. A threshold of 0.7 was used to define suitability, such that locations with a probability of occurrence <0.7 were set to 0 and locations with a probability ≥0.7 were set to 1. Binary predictions are commonly used to estimate how future species ranges will be affected. Grid cells with a value of 1 indicate potentially suitable habitats for *Zelkova schneideriana*, whereas cells with a value of 0 are considered unsuitable due to inappropriate environmental conditions for the species’ growth. Subsequently, water, barren, cropland, and urban were identified as unsuitable for afforestation in both the current and future scenarios and were thus excluded from the suitable distribution range. The resulting areas define the potential habitat for *Zelkova schneideriana* [33].

To examine the anticipated direction of range shifts for each species, we calculated the latitudinal and elevational changes in the distribution range expansion and contraction of *Zelkova schneideriana* for both current and future periods. Firstly, we extracted the latitude and elevation values of each pixel within the expanding and contracting distribution ranges of *Zelkova schneideriana*. Then, a Mann–Whitney U test was conducted to analyze the differences between the two periods, with effect size employed as supplementary evidence due to the large sample size. All data extraction and analyses were performed using R 4.2.3.

In this study, Fragstats 4.2 software was used to calculate twelve landscape pattern indices to better characterize the internal structure of the distribution range of *Zelkova schneideriana* under current and future climate conditions. These twelve indices specifically include the area-weighted mean patch area (AREA_AM), standard deviation of patch area (AREA_SD), patch density (PD), number of patches (NP), area-weighted mean fractal dimension (FRAC_AM), area-weighted mean euclidean nearest-neighbor distance (ENN_AM), standard deviation of euclidean nearest-neighbor distance (ENN_SD), standard deviation of fractal dimension (FRAC_SD), area-weighted mean shape index (SHAPE_AM), standard deviation of shape index (SHAPE_SD), aggregation index (AI), and landscape division index (DIVISION). The calculation formulas and descriptions for each landscape index can be found on the official website of Fragstats 4.2 (https://www.fragstats.org.).

## 5. Conclusions

This study successfully reveals the changes in the distribution of *Zelkova schneideriana* under the SSP1-2.6 and SSP5-8.5 climate scenarios by ensemble species distribution models and Fragstats software. It clarifies the impacts of climate and land-use changes on the distribution of *Zelkova schneideriana*. In the future, the distribution of *Zelkova schneideriana* will not only face contraction but also fragmentation, with these challenges being more pronounced under the SSP5-8.5 climate scenario. Temperature emerges as the most important factor influencing the distribution of *Zelkova schneideriana*, and the occupation of non-forest areas will severely affect its distribution. Furthermore, incorporating monitoring and management into conservation planning is necessary to allow for flexible responses to unforeseen changes in habitat structure and quality. The findings of this study emphasize the immediate need for protective measures to mitigate the impact of climate change and habitat fragmentation on *Zelkova schneideriana* and similar key species. Focusing solely on changes in distribution areas is insufficient; monitoring and protecting the genetic diversity of *Zelkova schneideriana* communities should also be prioritized. It is hoped that this study will serve as a reference and inspiration for future research on the effects of climate and land-use changes on plant distribution and quantification.

## Figures and Tables

**Table 1:** Results of the validation of TSS and AUC values for the model.

Metric	Sensitivity	Specificity	Calibration
TSS	96.474	83.997	0.805
AUC	96.513	84.936	0.956

**Table 2:** Relative contribution rate of bioclimatic variables.

Environment Variables	Relative Contribution
Bio6	50.5%
Bio2	28.5%
Bio15	13.4%
Elevation	7.6%

**Table 3:** Numerical characteristics of climatic variables in the current distribution area of *Zelkova schneideriana*.

Scenario	Time	Code	95% Quantiles		Mean	Units
			2.5%	97.5%		
Current	2000s	Bio6	−5.465	6.353	0.796	°C
SSP1-2.6	2021–2040	Bio6	−3.903	7.300	2.060	
	2041–2060	Bio6	−3.605	8.000	2.561	
	2061–2080	Bio6	−3.600	7.903	2.701	
	2081–2100	Bio6	−3.102	8.600	3.449	
SSP5-8.5	2021–2040	Bio6	−2.903	7.502	2.672	
	2041–2060	Bio6	−1.700	8.603	3.751	
	2061–2080	Bio6	−0.303	9.503	4.807	
	2081–2100	Bio6	−0.500	10.000	4.985	
Current	2000s	Bio2	10.250	6.548	8.116	°C
SSP1-2.6	2021–2040	Bio2	10.367	6.773	8.424	
	2041–2060	Bio2	10.650	6.991	8.699	
	2061–2080	Bio2	10.483	6.658	8.389	
	2081–2100	Bio2	10.517	6.374	8.243	
SSP5-8.5	2021–2040	Bio2	10.167	6.542	8.116	
	2041–2060	Bio2	10.425	6.841	8.425	
	2061–2080	Bio2	10.450	6.816	8.499	
	2081–2100	Bio2	10.842	7.308	8.971	
Current	2000s	Bio15	85.917	46.595	62.784	mm
SSP1-2.6	2021–2040	Bio15	87.016	49.086	64.053	
	2041–2060	Bio15	85.576	49.588	63.162	
	2061–2080	Bio15	89.904	46.841	65.417	
	2081–2100	Bio15	86.647	44.537	63.900	
SSP5-8.5	2021–2040	Bio15	87.585	47.330	63.172	
	2041–2060	Bio15	90.637	52.320	67.198	
	2061–2080	Bio15	86.172	45.350	64.493	
	2081–2100	Bio15	90.718	51.110	68.389	

## Data Availability

Data are contained within the article.
